# Efficacy of cardiometabolic drugs in reduction of epicardial adipose tissue: a systematic review and meta-analysis

**DOI:** 10.1186/s12933-023-01738-2

**Published:** 2023-01-31

**Authors:** Veronika A. Myasoedova, Valentina Parisi, Donato Moschetta, Vincenza Valerio, Maddalena Conte, Ilaria Massaiu, Michele Bozzi, Fabrizio Celeste, Dario Leosco, Guido Iaccarino, Stefano Genovese, Paolo Poggio

**Affiliations:** 1 Unit for the Study of Aortic, Valvular, and Coronary Pathologies, Centro Cardiologico Monzino IRCCS, Via Carlo Parea 4, 20138 Milan, Italy; 2 Department of Translational Medical Sciences, Federico II University, Naples, Italy; 3 Casa Di Cura San Michele, Maddaloni, Italy; 4 Department of Advanced Biomedical Sciences, Federico II University, Naples, Italy

## Abstract

**Background:**

Epicardial adipose tissue (EAT) plays an important role in cardiometabolic risk. EAT is a modifiable risk factor and could be a potential therapeutic target for drugs that already show cardiovascular benefits**.** The aim of this study is to evaluate the effect of cardiometabolic drugs on EAT reduction.

**Methods:**

A detailed search related to the effect on EAT reduction due to cardiometabolic drugs, such as glucagon-like peptide-1 receptor agonist (GLP-1 RA), sodium-glucose cotransporter-2 inhibitors (SGLT2-i), and statins was conducted according to PRISMA guidelines. Eighteen studies enrolling 1064 patients were included in the qualitative and quantitative analyses.

**Results:**

All three analyzed drug classes, in particular GLP-1 RA, show a significant effect on EAT reduction (GLP-1 RA standardize mean difference (SMD) = − 1.005; p < 0.001; SGLT2-i SMD = − 0.552; p < 0.001, and statin SMD = − 0.195; p < 0.001). The sensitivity analysis showed that cardiometabolic drugs strongly benefit EAT thickness reduction, measured by ultrasound (overall SMD of − 0.663; 95%CI − 0.79, − 0.52; p < 0.001). Meta-regression analysis revealed younger age and higher BMI as significant effect modifiers of the association between cardiometabolic drugs and EAT reduction for both composite effect and effect on EAT thickness, (age Z: 3.99; p < 0.001 and Z: 1.97; p = 0.001, respectively; BMI Z: − 4.40; p < 0.001 and Z: − 2.85; p = 0.004, respectively).

**Conclusions:**

Cardiometabolic drugs show a significant beneficial effect on EAT reduction. GLP-1 RA was more effective than SGLT2-i, while statins had a rather mild effect. We believe that the most effective treatment with these drugs should target younger patients with high BMI.

**Graphical Abstract:**

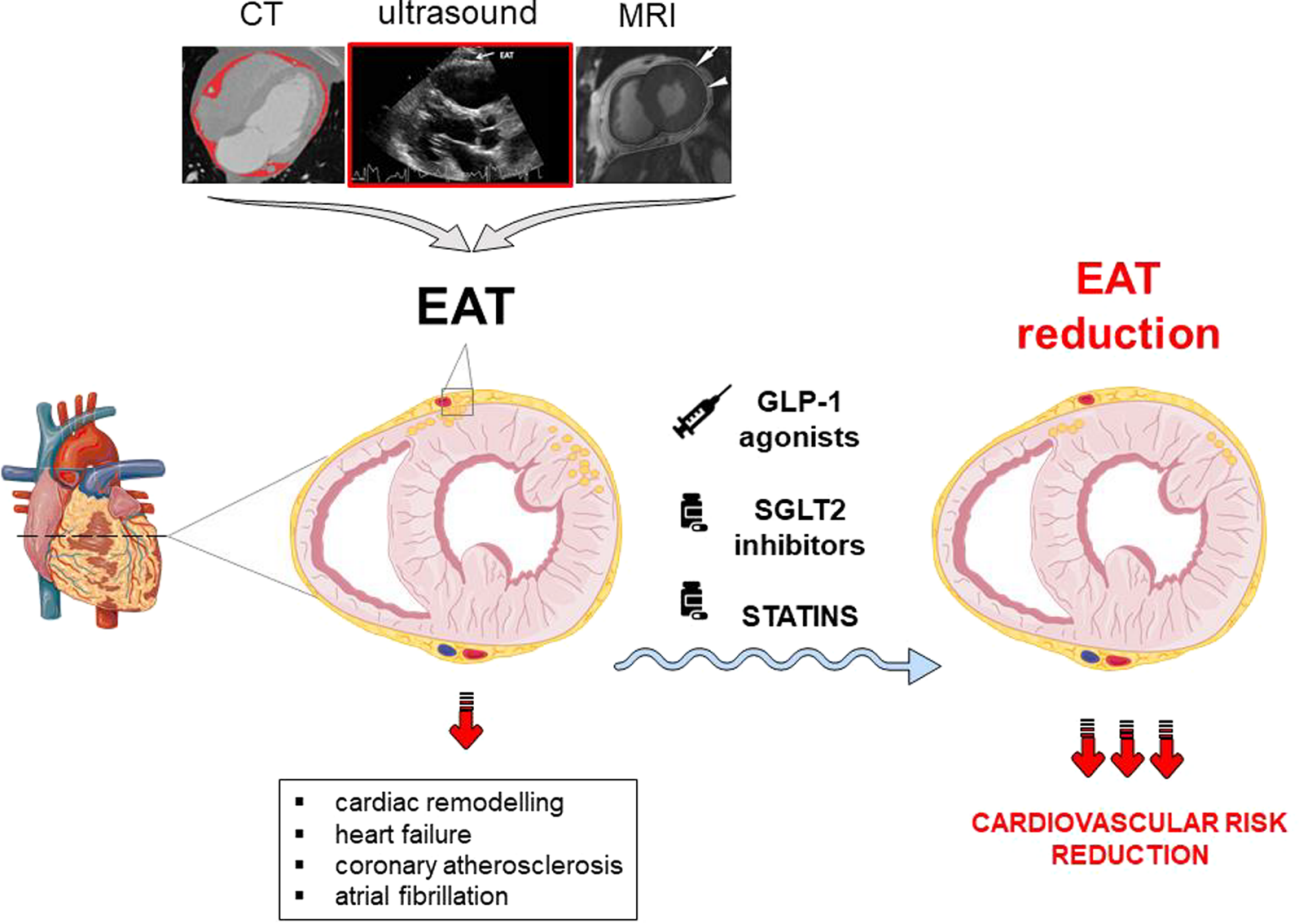

**Supplementary Information:**

The online version contains supplementary material available at 10.1186/s12933-023-01738-2.

## Introduction

Cardiac remodeling is the typical feature of chronic heart failure [1] and several pathophysiological conditions, such as acute/chronic ischemia, hypertension, and diabetes. However, the pathological mechanisms involved are not entirely elucidated [2].

Epicardial adipose tissue (EAT) is anatomically connected to the myocardium and it has been suggested as a potential player in the development of cardiac remodeling [3, 4], as well as contributing to coronary artery disease [5] and atrial fibrillation [6]. EAT can affect cardiac function through increased inflammation, fibrosis, and autonomic dysregulation [7–10]. In addition, increased EAT volume can impair ventricular relaxation through a mechanical effect, as observed in obesity [11].

EAT is a modifiable risk factor that can be assessed by different cardiac imaging techniques such as echocardiography, magnetic resonance imaging (MRI), and cardiac computed tomography (CT) [12]. Non-invasive quantification of EAT can help to predict and stratify cardiovascular risk [13, 14]. Indeed, recent evidence suggested that EAT predicts cardiovascular disease and mortality incidence in patients with type 2 diabetes [15]. EAT could also be a potential therapeutic target for drugs that already show cardiovascular benefits, particularly glucose-lowering drugs, such as glucagon peptide-1 agonist (GLP-1 RA) and sodium-glucose co-transporter-2 inhibitors (SGLT2-i), as well as lipid-lowering drugs (*i.e.*, statins), that already have shown the ability to reduce the incidence of major cardiovascular events [16–19]. The pleiotropic effects of these cardiometabolic drugs have recently been reported in several studies [13]. However, a comprehensive analysis of EAT modifications due to cardiometabolic drugs has not been performed yet.

Our review and meta-analysis aimed to clarify: (1) the therapeutic effect of GLP-1 RA, SGLT2-i, and statin on EAT reduction; (2) the effect of cardiometabolic drugs on echocardiographic EAT thickness; (3) the time-dependent effect of cardiometabolic drugs on EAT reduction; and (4) the impact of patient baseline clinical characteristics on the association between cardiometabolic drugs and EAT reduction.

## Methods

### Data sources and searches

A detailed protocol for the search strategy of this review was developed prospectively, specifying objectives, study selection criteria, outcomes, and statistical methods.

To detect all available studies on the association between cardiometabolic drugs effect and EAT a systematic search was evaluated in the electronic databases (PubMed, Web of Science, and Scopus) according to guidelines [20]. The search string applied to PubMed was the following: (Epicardial Adipose Tissue OR EAT) AND (thickness OR volume) AND (glucagon-like peptide 1 OR GLP-1 OR GLP1 OR GLP1-agonist OR sodium glucose cotransporter 2 OR sodium–glucose co-transporter-2 OR SGLT2 OR SGLT-2 OR SGLT2-Inhibitors) AND (statin). The last search was performed in June 2022.

### Study selection, data extraction, and quality assessment

According to the stipulated protocol, all studies reporting data about the association between the effect of cardiometabolic drugs, such as GLP-1 RA, SGLT2-i, and statins, on EAT were included. Case reports, reviews, and articles on animal models were excluded. Studies in which the effect of different cardiometabolic drugs on EAT was assessed by ultrasonography, CT, and/or MRI at baseline and follow-up were included in the analysis. Studies with more than one compound of the same drug type were evaluated separately, in particular statin [21, 22] and GLP-1 RA [23]. In each study, data regarding major clinical and demographic characteristics in patients with EAT reduction under different cardiometabolic drugs were extracted. Three types of analysis were performed: (1) a combination of different diagnostic approaches, such as ultrasound, CT, and MRI, applied for EAT assessment; (2) evaluation of the effect of cardiometabolic drugs on EAT thickness assessed only by one imaging technique (i.e., ultrasound, measured in mm); (3) assessment of the time-dependent effect of cardiometabolic drug assumption on EAT at 3 and 6 months of follow-up.

### Statistical analysis and risk of bias assessment

Statistical analysis was performed using Comprehensive Meta-analysis Version 3.3.070 (Biostat, Englewood, NJ 2014). The differences in continuous variables were expressed as a standardized mean difference (SMD) and 95% confidence intervals (CI). The overall effect was tested using Z scores and significance was set at p < 0.05. Statistical heterogeneity among studies was assessed with chi-square Cochran’s Q test and with I^2^ statistic, which measures the inconsistency across study results and describes the proportion of total variation in study estimates, that is due to heterogeneity rather than sampling error. In detail, I^2^ values of 0% indicate no heterogeneity, 25% low, 25–50% moderate, and 50% or more high heterogeneity [24].

The evaluation of the methodological quality of each study was performed accordingly Newcastle–Ottawa Scale (NOS). The scoring system encompasses three major domains (selection, exposure, outcome) and a resulting score range between 0 and 9, a higher score representing a better methodological quality. Moreover, the reference lists of all included articles were manually consulted. Two authors (VAM and VP) analyzed each article and performed the data extraction separately. In case of disagreement, a third investigator was consulted (PP). Discrepancies were resolved by consensus. Egger's test and funnel plots of the logit event rate *vs* the standard error were used as a graphical representation to evaluate the risk of bias. To assess the small-study effect, funnel plots were visually inspected for asymmetry and Egger's test was used to assess publication bias, over and above any subjective evaluation, with p < 0.05 being considered statistically significant [25]. In order to be as conservative as possible, the random-effect method was used for all analyses to consider the variability among the included studies [26]. In the case of significant publication bias, Duval and Tweedie’s trim and fill method was used to allow for the estimation of adjusted effect size [26].

### Meta-regression analyses

In order to assess the impact of demographic (mean age and sex) and clinical [body mass index (BMI), low-density lipoprotein (LDL-C), hemoglobin A1c (HbA1c)] characteristics on the EAT reduction in patients under cardiometabolic drug treatment, we performed meta-regression analyses after implementing regression models with the rate of EAT reduction as dependent variable (y) and the above-mentioned covariates as independent variables (x) [27].

## Results

**Fig. 1:**
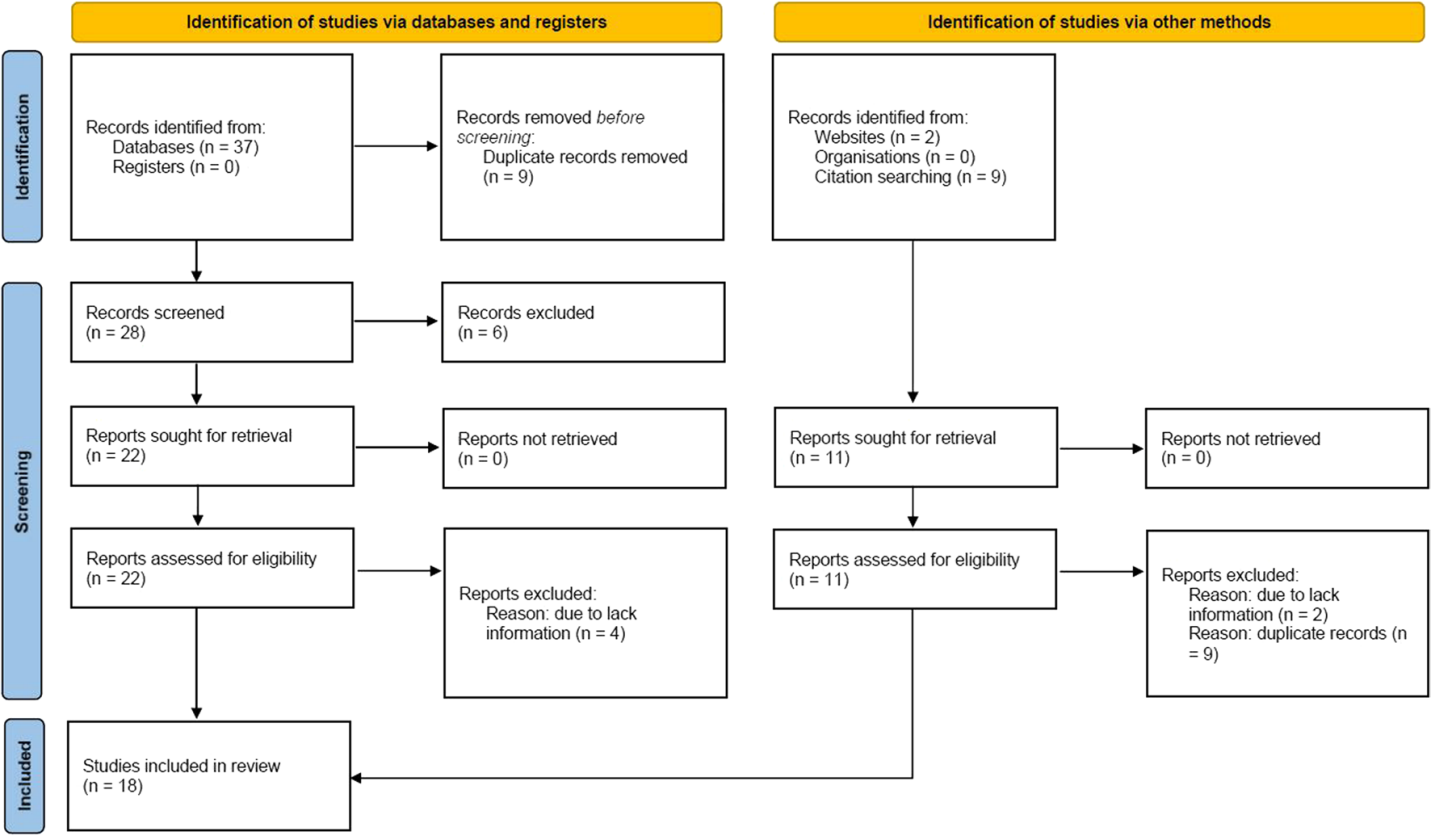
Prisma Flow Chart. The flow chart represents the number of studies evaluated according to PRISMA guidelines

The search strategy identifies 30 articles, Fig. 1. Duplicate results were excluded, and after a screening of the titles and the abstracts, twenty-two articles were selected for full-text evaluation. The revision of full-length articles allowed the exclusion of four studies due to irrelevant information in their content. Overall, eighteen studies [21–23, 28–43] enrolling 1064 patients were included in the qualitative and quantitative analyses of the effect of the cardiometabolic drug on EAT.

Data on GLP-1 RA were reported in seven studies on 240 patients and eight studies reported data on SGLT2-1, including 221 patients. In addition, three studies show the effect of statins on EAT in 603 patients. Eight studies included the data from randomized clinical trials (RCT), while ten studies represent the data from single-arm studies (SAS). A total of 50% of included patients were females, with a mean age of 57.5 years (range: 43–67 years). The mean BMI was 31.2 kg/m^2^ (range: 22.6–37.8 kg/m^2^), the mean LDL-C was 111 mg/dL with mean LDL-C ∆ of − 9.9%, and the mean HbA1c was 7.5% (58 mmol/mol) with mean HbA1c reduction of − 9.3% (only in patients under SGLT2-i and GLP-1 RA treatment). The mean follow-up was 5.2 ± 2.6 months. Results of the NOS quality assessment are reported in Additional file 2: Table S1.

**Fig. 2:**
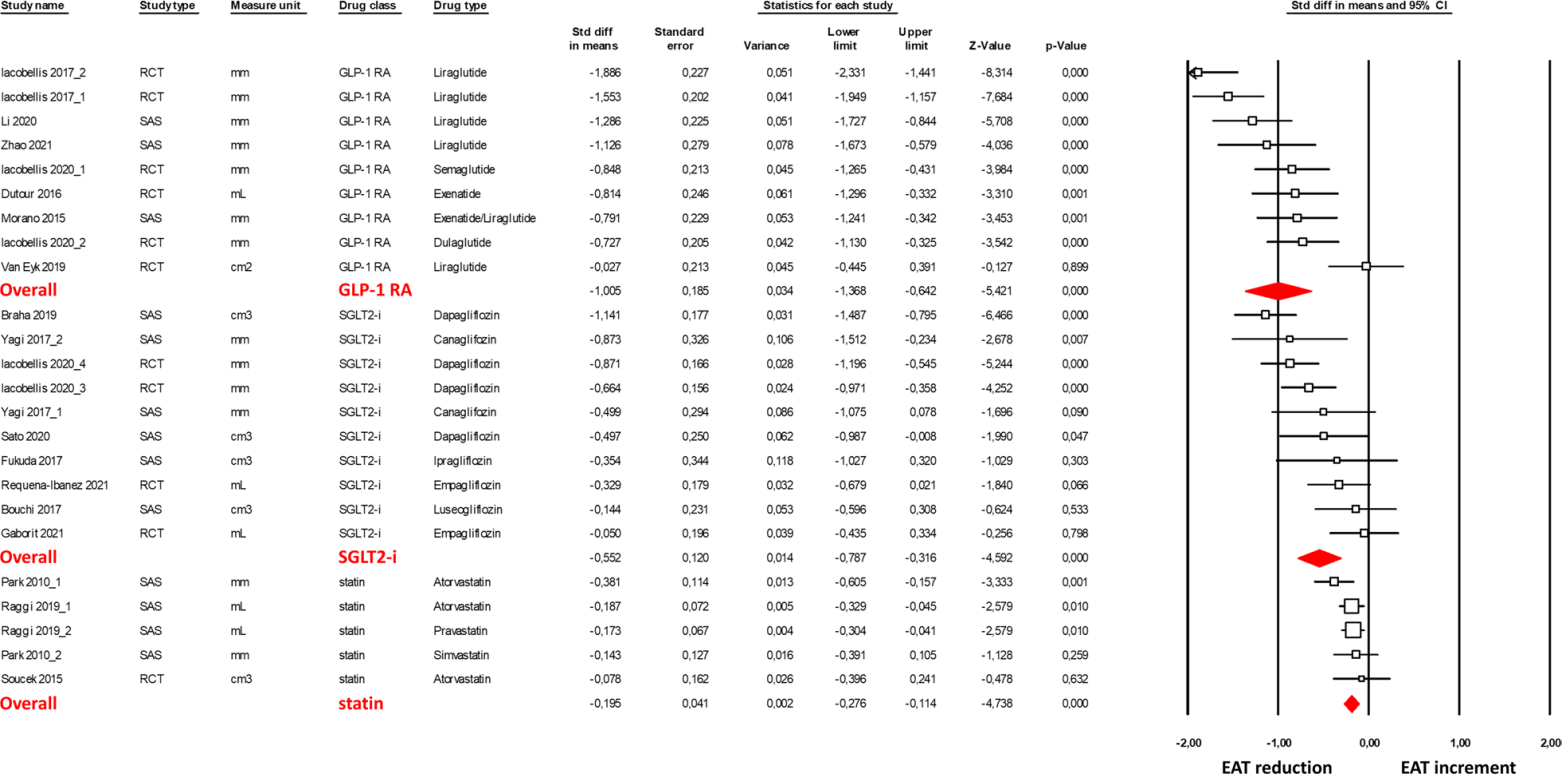
Forest plots of the cardiometabolic drug effect on the epicardial adipose tissue (EAT) reduction, assessed by ultrasound, computed tomography and magnetic resonance imaging. EAT reduction was evaluated with the standardized difference in means (SMD) between baseline and follow-up. The diamond represents the estimated overall effect, while the squares represent each study with 95% CI

The effect of cardiometabolic drugs on EAT is shown in Fig. 2 as SMD. This method was implemented to combine different diagnostic approaches, such as ultrasound, CT, and MRI applied for EAT assessment. All three analyzed drug classes show a significant effect on EAT reduction (GLP-1 RA, SMD = − 1.005, 95% CI − 1.37, − 0.64, p < 0.001; SGLT2-1, SMD = − 0.552; 95% CI − 0,79, − 0.32, p < 0.001; and statins, SMD = -0.195, 95% CI − 0.79 − 0.32, p < 0 0.001).

The sensitivity analysis indicated a significantly stronger effect of GLP-1 RA on the EAT reduction compared to SGLT2-i (~ 2 folds, p = 0.04). In addition, considering the difference in follow-up times in the analyzed studies, the sensitivity analysis showed a significant reduction in EAT at both 3 and 6 months of follow-up (SMD = − 0.698, 95% CI − 0.89, − 0.50, p < 0.001 and SMD = − 0.757, 95% CI − 1.06, − 0.45, p < 0 0.001, respectively; Additional file 2: Figures S1 and S2). Interestingly, after 6 months of drug intake, the effect of GLP-1 RA and SGLT2-i on EAT reduction was similar.

The heterogeneity among the studies was significant for GLP-1 RA (I^2^: 83.7%, p < 0.001) and SGLT2-i (I^2^: 67.6%, p = 0.001), while there was no heterogeneity among statin (I^2^: 0.00%, p = 0.483) studies. In contrast, the examination of funnel plots of effect size versus standard error for the studies evaluating the different effects of cardiometabolic drugs on EAT reduction shows the absence of publication bias, confirmed by Egger’s test for GLP-1 RA (p = 0.840), for SGLT-2 inhibitors (p = 0.487), and statin (p = 0.954).

**Fig. 3:**
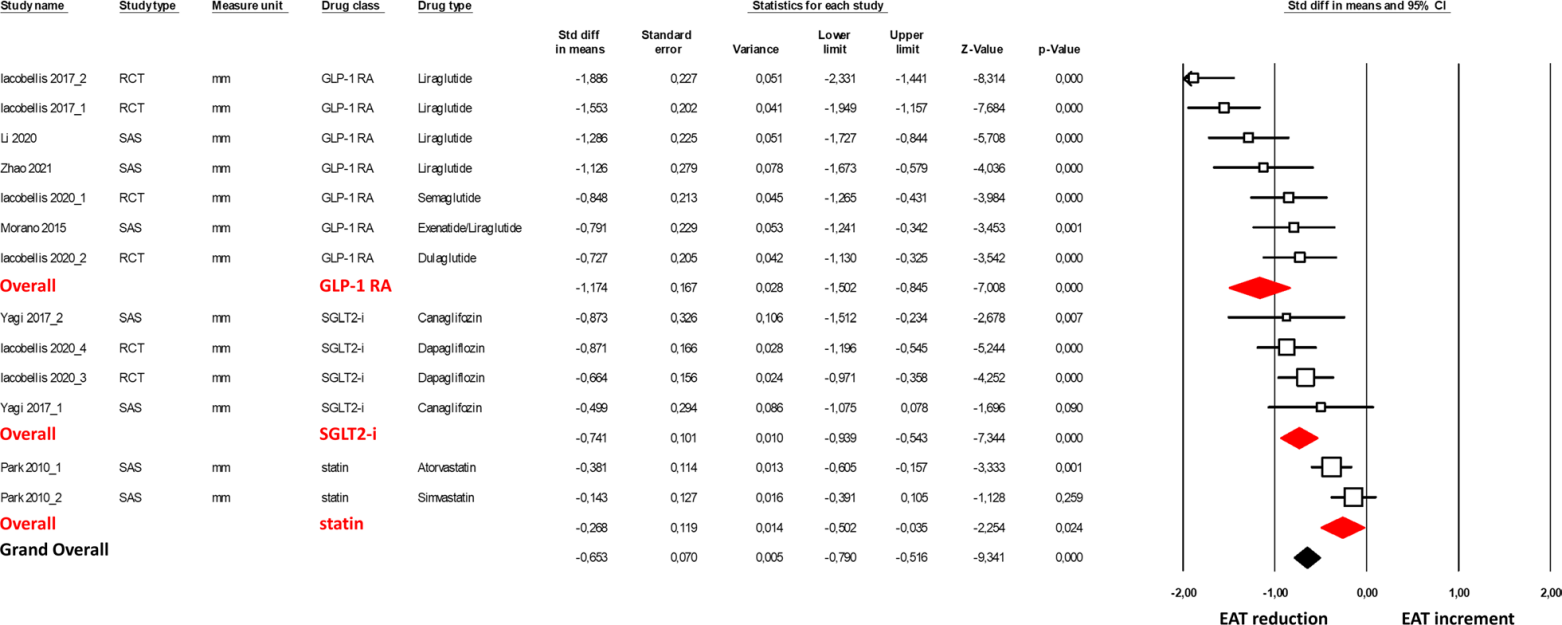
Forest plots the effect of cardiometabolic drug on the epicardial adipose tissue thickness reduction assessed by ultrasound. EAT thickness reduction was evaluated with the standardized difference in means (SMD) between baseline and follow-up. The diamond represents the estimated overall effect, while the squares represent each study with 95% CI

To further corroborate our findings, we evaluated the effect of cardiometabolic drugs on EAT thickness assessed only by one imaging technique (*i.e.*, ultrasound, measured in mm). Echocardiographic evaluation of EAT thickness was performed as a perpendicular measurement on the free wall of the right ventricle at the end-systole in 3 cardiac cycles. The parasternal long-axis view allowed for the most accurate measurement of EAT on the right ventricle, with optimal cursor beam orientation in each view. The meta-analysis was thus performed on eight studies [21, 23, 28, 29, 34, 36–38] (Fig. 3). The obtained results showed a significant EAT thickness reduction under GLP-1 RA, SGLT2-i, and statin treatments with an overall SMD of − 0.663 (95% CI − 0.79, − 0.52, p < 0.001). Again, the sensitivity analysis confirmed a significantly higher EAT thickness reduction under GLP-1 RA treatment compared to SGLT2-i (p = 0.03).

A separate sensitivity analysis at 3 and 6 months of follow-up was also performed for studies that included EAT echocardiographic assessment only. A significant overall effect of GLP-1 RA and SGLT2-i on EAT thickness reduction was observed for both 3 and 6 months of follow-up (SMD = -− 0.836, 95% CI − 1.03, − 0.64, p < 0 0.001; SMD = − 1.217, 95% CI − 1.91, − 0.52, p = 0 0.001, respectively; Additional file 2: Figs. S3-S4).

A high heterogeneity among the studies was observed only in GLP-1 RA studies (I^2^: 74.5%, p < 0.001). Overall, funnel plots of effect size versus standard error for the overall effect of cardiometabolic drugs on EAT thickness showed an asymmetric distribution and the Egger’s test confirmed the presence of a significant publication bias (Egger’s p = 0.023, Additional file 2: Fig. S5). After adjusting for publication bias (Duval and Tweedie’s trim and fill analysis), results were confirmed with an estimated point for SMD of − 0.95 (95% CI − 1.27, − 0.64).

**Fig. 4:**
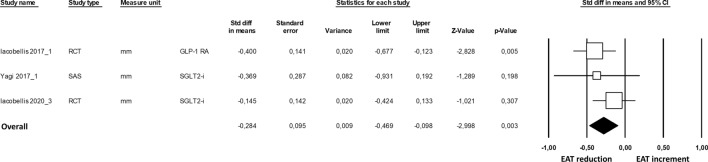
Forest plots the effect of cardiometabolic drug on the epicardial adipose tissue thickening at 3- and 6-months follow-up. EAT thickness reduction was evaluated with the standardized difference in means (SMD) between 3 and 6 months of follow-up. The diamond represents the estimated overall effect, while the squares represent each study with 95% CI

The time-dependent effect of cardiometabolic drug assumption was analyzed in three studies [29, 37, 38], which report the results on EAT thickness reduction at 3 and 6 months in the same study. The results of the meta-analysis suggested significantly higher EAT thickness reduction at 6 months of GLP-1 RA or SGLT2-i administration (SMD = − 0.284, 95% CI − 0.47, − 0.10, p = 0.003; Fig. 4), without heterogeneity (I^2^: 0.00%, p = 0.424) among studies and publication bias (p = 0.850).

**Fig. 5:**
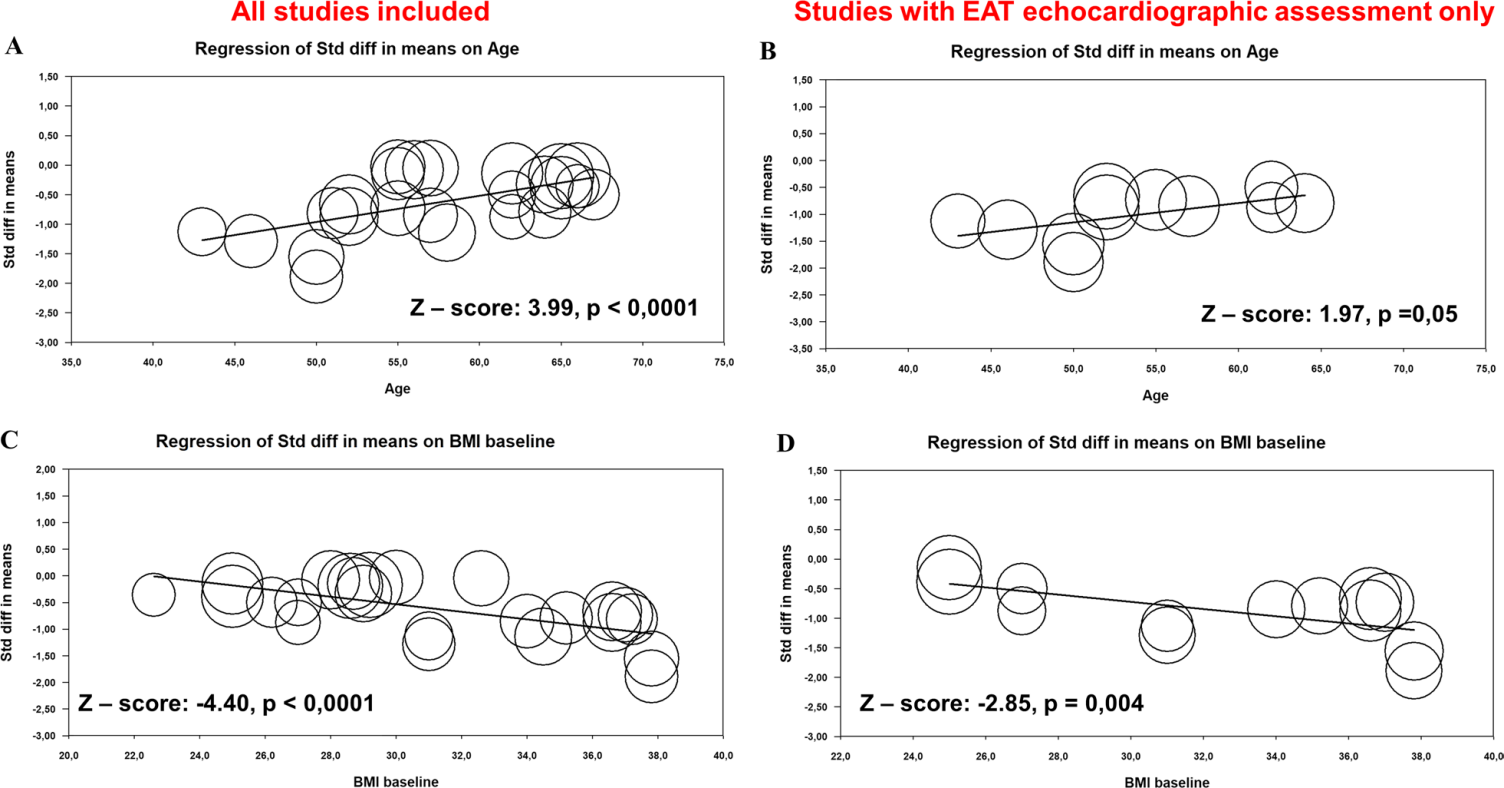
Meta-regression analysis. Impact of age on the difference in composite cardiometabolic drugs effect and EAT reduction (**A**), and cardiometabolic drugs effect and EAT thickness reduction measured ultrasound (**B**). Impact of BMI on the difference in composite cardiometabolic drugs effect and EAT reduction (**C**), and cardiometabolic drugs effect and EAT thickness reduction measured ultrasound (**D**)

Finally, we employed a meta-regression analysis approach to evaluate which significant effect modifiers existed in the association between cardiometabolic drugs and EAT reduction. Our results revealed that cardiometabolic drug intake benefitted younger patients with a high BMI, both in all included studies and in studies only with EAT echocardiographic measurement (Fig. 5). No associations were observed regarding the effect of sex, LDL-C levels, and HbA1c levels (Additional file 2: Fig. S6).

## Discussion

Results of our meta-analysis consistently show that all analyzed drug types, GLP-1 RA, SGLT2-i, and statins, significantly reduced EAT. The greatest efficacy was observed in patients treated with GLP-1 RA compared with SGLT2-i or statins. However, after 6 months of drug intake, the effect of GLP-1 RA and SGLT2-i was similar. In addition, cardiometabolic drugs showed a significant effect on EAT thickness measured by ultrasound, both at 3- and 6-month follow-up, with a greater effect in patients on longer intake. Younger age and higher BMI were associated with a stronger effect of cardiometabolic drugs on EAT reduction.

EAT, a lipid storage depot covers the surface of the heart and surrounds the coronary arteries while also functioning as an endocrine organ secreting hormones and releasing pro-inflammatory cytokines [44]. In addition, EAT is believed to have paracrine and autocrine activities that may induce atherosclerosis and myocardial fibrosis [45]. Excessive EAT was shown to be associated with cardiometabolic risk, and fatal and non-fatal coronary events [46]. Thus, reducing EAT harmful sequela could help in preventing the above-mentioned cardiovascular diseases.

Although the positive effect of lifestyle changes (diet and exercise) and bariatric surgery on EAT has been previously demonstrated [47–49], no pharmacological tools specifically developed for EAT reduction are available yet. To date, the effects of lipid-lowering and glucose-lowering drugs have been tested as possible therapeutic approaches to reduce EAT.

Intensive statin therapy showed a beneficial, dose-dependent effect on EAT reduction [50], independently of their lipid-lowering effects [22]. Parisi et al*.* [51] suggested that this pleiotropic effect of statin on EAT is due to its anti-inflammatory properties. Furthermore, a statin-induced reduction in EAT thickness can potentially be due to the modulation of peroxisome proliferator-activated receptors (PPARs) [13]. However, in our meta-analysis, we observed a significant but rather mild effect of statins on EAT reduction (weighted EAT average reduction of 6%). Interestingly, a recent type of lipid-lowering drug, proprotein convertase subtilisin/kexin 9 (PSCK9) inhibitors, showed a more effective reduction in EAT after 6 months of intake (~ 20%), again, independently of LDL-C [52]. Thus, the use of lipid-lowering drugs is helpful to reduce EAT, acting on inflammation. However, a deeper understanding of the underlying mechanism of lipid-lowering drug's action on EAT is needed.

Glucose-lowering drugs, such as SGLT2-i and GLP-1 RA, have been shown to have a cardioprotective effect independent of blood glucose-lowering [53, 54]. Thus, they could be considered pharmacological targets in the treatment of cardiometabolic disorders. Indeed, SGLT2-i and GLP-1 RA can target both left atrial and coronary EAT for the treatment and prevention of atrial fibrillation and coronary artery disease by reducing EAT inflammation and increasing free fatty acid oxidation [13].

Regarding SGLT2-i, it has been shown that EAT volume could be reduced improving also inflammation and body weight [29, 40, 43]. In addition, the reduction of EAT by SGLT2-i led to remission of diastolic dysfunction [42] and improvement of conduction velocity within the atria [32]. Diaz-Rodriguez et al*.* [55] found that the use of SGLT2-i was associated with an increase in EAT glucose uptake, reduced secretion of proinflammatory chemokines, and improved differentiation ability. Results of a very recent study demonstrated that the SGLT2-i improves not only EAT but even interstitial myocardial fibrosis, aortic stiffness, and inflammation markers also in non-diabetic patients with heart failure with reduced ejection fraction [33]. Our meta-analysis is in line with recent studies showing a significant beneficial effect of SGLT2-i on EAT.

Interestingly, our analysis of GLP-1 RA showed a two-times stronger effect on EAT reduction compared to SGLT2-i. GLP-1 RA is characterized by rapid (within 3 months), large (more than 25% weighted average), and dose-dependent effects on EAT reduction [37, 38]. The mechanisms, explaining the independent and marked role of GLP-1 RA on EAT reduction, are not clearly understood. However, activation of EAT GLP-1 receptors was shown to be associated with inducing fat browning (white into brown fat differentiation of pre-adipocytes, improving myocardial insulin sensitivity), thereby improving myocardial metabolism [56], suggesting that these metabolic changes might contribute to the beneficial effect of GLP-1 RA on the cardiovascular system [13, 57]. In addition, it cannot be excluded that these beneficial consequences could also be due to their antioxidant and anti-inflammatory effects [58].

Of note, both SGLT2-i and GLP-1 RA show a significant rapid effect, within 12 weeks [37, 38], on EAT reduction, while results of our meta-analysis indicated that prolonged intake, beyond 24 weeks, could lead to more pronounced and stable beneficial effects.

Above and beyond, EAT could be considered a modifiable risk marker, and weight loss induced by diet, exercise, or drug treatment plays an important role in reducing EAT, and consequently cardiovascular risk [59]. Results of our meta-regression analysis indicate a strong association between EAT reduction and BMI in patients under cardiometabolic drug treatment, in particular, patients with higher BMI levels had more robust EAT reduction. In addition, younger age was found to be a significant effect modifier in the association between cardiometabolic drugs effect and EAT. Therefore, special attention should be paid to risk characterization and management of patients with increased EAT at a young age and high BMI. This finding can be surprising since the known association of EAT with age [60]. The greater EAT reduction in younger patients with higher BMI suggests that medical therapy can be more effective in high-risk patients without established cardiovascular diseases. Indeed, the effects of drug therapies on EAT reduction may be more evident in absence of other factors that are strongly associated to EAT increase (*e.g.*, older age, coronary artery disease, etc.). This hypothesis could also in part explain the lower effect of statins on EAT reduction highlighted by this meta-analysis, as studies exploring the effects of statins often include older patients with established coronary artery disease.

Another aspect that needs to be discussed is the large imaging techniques variability of EAT assessment. Cardiac multidetector CT and MRI provide volumetric measurements of EAT and additional functional information, such as EAT density or fat attenuation index, which correlated with perivascular adipose tissue inflammation, or intramyocardial lipid content assessed by MRI [13]. However, these valuable techniques are minimally invasive, not readily available, and not cost-effective. Echocardiographic measurement of EAT thickness is still the most used technique with several advantages, including low cost, accessibility, and reproducibility [13]. However, in echocardiography, EAT measurements are very variable among different studies. EAT is described both as an echo-free space and/or a hyperechoic tissue and measured either at end-diastole [61, 62] or end-systole [63, 64]. Importantly, a uniform standardized method for EAT quantification is still lacking and normal range values remain to be established [65, 66].

Therefore, considering all the evidence, we may suppose that GLP-1 RA and SGLT2-i, due to their cardiovascular beneficial effect, can be considered as potential therapeutic tools to positively modify EAT thickness/volume and functional behavior, probably restoring EAT homeostasis even in patients without diabetes. In addition, EAT monitoring, particularly echocardiographic assessment of EAT thickness, is a quick and simple examination that already takes a part of the clinical routine, allowing effective monitoring of therapy over time and proper management of cardiovascular patients.

### Study limitations

Our study has the following potential limitations. First, our systematic review and meta-analysis was not prospectively registered as suggested by the Cochrane guidelines. Nevertheless, we followed the latest Additional file 1 PRISMA guidelines (2020). Second, in the analyzed publications, the diagnosis of EAT was made by different imaging techniques, however, standardized mean difference (SMD) was used to combine different diagnostic approaches, such as ultrasound, computed tomography, and magnetic resonance imaging. Third, statistical heterogeneity among the studies was high, yet, the results remained robust when performing sensitivity analysis. Last, the follow-up periods were different between all the analyzed studies, nevertheless, when analyzing the effect of cardiometabolic drugs separately, at 3- and 6- months of follow-up, the EAT reduction remained significant. Additional file: As per journal requirements, every additional file must have a corresponding caption. In this regard, please be informed that the caption of Additional file [2]was taken from the additional e-file itself. Please advise if action taken appropriate and amend if necessary. The action taken is appropriate. Thank you.

## Conclusion

All studied cardiometabolic drugs were associated with a significant beneficial effect on EAT reduction. GLP-1 RA in comparison with SGLT2-i showed stronger changes in EAT, while statins had a rather mild beneficial effect. Both SGLT2-i and GLP-1 RA show a significant and rapid effect on EAT thickness within 3 months, but prolonged intake beyond 6 months led to a more pronounced and stable beneficial effect. Cardiometabolic drugs, particularly GLP-1 RA and SGLT2-i, may be considered as a potential therapeutic target in cardiovascular prevention acting also on dysfunctional EAT, with a focus on young patients with a high BMI. Nevertheless, further studies are needed to standardize the methods of quantification and identification of normal EAT values as well as the understanding of cellular and molecular mechanisms involved in EAT dysregulation.

## Supplementary Information

**Additional file 1.** Preferred Reporting Items for Systematic Reviews and Meta-Analyses (PRISMA) 2020 Checklist.

**Additional file 2: Table S1. **Quality assessment with Newcastle-Ottawa scale of the included studies. **Figure S1. **Forest plots of the GLP-1 agonists and SGLT-2 inhibitor's effect on epicardia adipose tissue (EAT) reduction within 3 months of follow-up. **Figure S2. **Forest plots of the GLP-1 agonists and SGLT-2 inhibitor's effect on EAT reduction within 6 months of follow-up. **Figure S3. **Forest plots of the GLP-1 agonists and SGLT-2 inhibitor's effect on EAT thickening reduction measured by ultrasound within 3 months of follow-up. **Figure S4. **Forest plots of the GLP-1 agonists and SGLT-2 inhibitor's effect on epicardia adipose tissue thickening reduction measured by ultrasound within 6 months of follow-up. **Figure S5. **Funnel plot of effect size versus standard error for the overall effect of cardiometabolic drugs on EAT thickening. Dots represent the single studies while diamonds are the overall standardized mean difference (Std diff in means). White filling refers to real studies while black fill relates to Duval and Tweedie’s trim and fill method. **Figure S6. Meta-regression analysis**. Impact of HbA1c on the difference in composite cardiometabolic drugs effect and EAT reduction (**A**), and cardiometabolic drugs effect and EAT thickness reduction measured ultrasound (**B**). Impact of cholesterol low-density lipoprotein levels (LDL-C) on the difference in composite cardiometabolic drugs effect and EAT reduction (**C**), and cardiometabolic drugs effect and EAT thickness reduction measured ultrasound (**D**). Impact of male sex on the difference in composite cardiometabolic drugs effect and EAT reduction (**E**), and cardiometabolic drugs effect and EAT thickness reduction measured ultrasound (**F**).

## Data Availability

The manuscript entirely reports published data. Spreadsheets with data as compiled for the present manuscript can be made available on reasonable request to the corresponding author.
