# Supplementary material for: Efficacy of cardiometabolic drugs in reduction of epicardial adipose tissue: a systematic review and meta-analysis
Source: Cardiovasc Diabetol. 2023 Jan 31;22:23. doi: 10.1186/s12933-023-01738-2 (PMC9890718; doi:10.1186/s12933-023-01738-2)
Supplement: Supplementary file 2 — Additional file 2: Table S1. Quality assessment with Newcastle-Ottawa scale of the included studies. Figure S1. Forest plots of the GLP-1 agonists and SGLT-2 inhibitor's effect on epicardia adipose tissue (EAT) reduction within 3 months of follow-up. Figure S2. Forest plots of the GLP-1 agonists and SGLT-2 inhibitor's effect on EAT reduction within 6 months of follow-up. Figure S3. Forest plots of the GLP-1 agonists and SGLT-2 inhibitor's effect on EAT thickening reduction measured by ultrasound within 3 months of follow-up. Figure S4. Forest plots of the GLP-1 agonists and SGLT-2 inhibitor's effect on epicardia adipose tissue thickening reduction measured by ultrasound within 6 months of follow-up. Figure S5. Funnel plot of effect size versus standard error for the overall effect of cardiometabolic drugs on EAT thickening. Dots represent the single studies while diamonds are the overall standardized mean difference (Std diff in means). White filling refers to real studies while black fill relates to Duval and Tweedie’s trim and fill method. Figure S6. Meta-regression analysis. Impact of HbA1c on the difference in composite cardiometabolic drugs effect and EAT reduction (A), and cardiometabolic drugs effect and EAT thickness reduction measured ultrasound (B). Impact of cholesterol low-density lipoprotein levels (LDL-C) on the difference in composite cardiometabolic drugs effect and EAT reduction (C), and cardiometabolic drugs effect and EAT thickness reduction measured ultrasound (D). Impact of male sex on the difference in composite cardiometabolic drugs effect and EAT reduction (E), and cardiometabolic drugs effect and EAT thickness reduction measured ultrasound (F). [file 12933_2023_1738_MOESM2_ESM.docx]

**Table S1. Quality assessment with Newcastle-Ottawa scale of the included studies.**

| **Author, year** | **SELECTION** | | | | **COMPARABILITY** | **OUTCOME** | | |  |
| --- | --- | --- | --- | --- | --- | --- | --- | --- | --- |
|  | **Representativeness of the Exposed Cohort** | **Selection of the Non-Exposed Cohort** | **Ascertainment of Exposure** | **Demonstration That Outcome of Interest Was Not Present at Start of Study** |  | **Assessment of Outcome** | **Enough Follow-Up Long for Outcomes to Occur** | **Adequacy of Follow Up of Cohorts** | **Quality** |
| Gaborit 2021 | * | * | * | - | ** | - | * | * | **7** |
| Requena-Ibanez 2021 | * | * | * | - | * | - | * | * | **6** |
| Iacobellis 2020_1 | ** | * | * | - | ** | - | * | * | **8** |
| Sato 2020 | * | * | * | - | ** | - | * | * | **7** |
| Braha 2020 | * | - | * | - | ** | - | * | * | **6** |
| Yagi 2017 | * | - | * | - | ** | - | ** | * | **7** |
| Bouchi 2017 | * | - | * | - | * | - | * | * | **5** |
| Fukuda 2017 | * | - | * | - | * | - | * | * | **5** |
| Zhao 2021 | * | - | * | - | * | - | * | * | **5** |
| Li 2020 | * | - | * | - | * | - | * | * | **5** |
| Iacobellis 2020_3 | * | - | * | - | ** | - | ** | * | **7** |
| Van Eyk 2019 | * | * | * | - | * | - | * | * | **6** |
| Iacobellis 2017 | ** | - | * | - | ** | - | ** | * | **8** |
| Dutour 2016 | * | - | * | - | * | - | * | * | **5** |
| Morano 2015 | * | - | * | - | * | - | * | * | **5** |
| Raggi 2019 | ** | - | * | - | * | - | * | * | **6** |
| Soucek 2015 | * | * | * | - | * | - | * | * | **6** |
| Park 2010 | ** | - | * | - | ** | - | * | * | **7** |


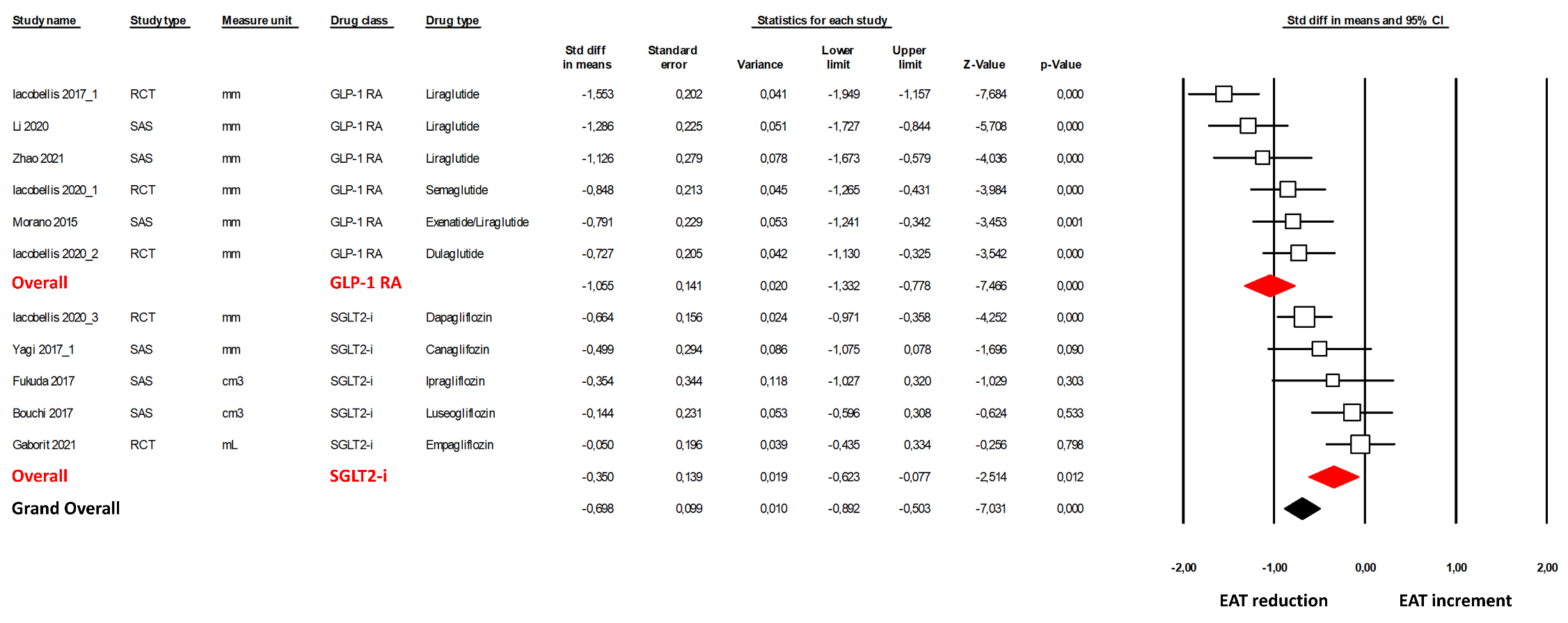


**Figure S1.** Forest plots of the GLP-1 agonists and SGLT-2 inhibitor's effect on epicardia adipose tissue (EAT) reduction within 3 months of follow-up.


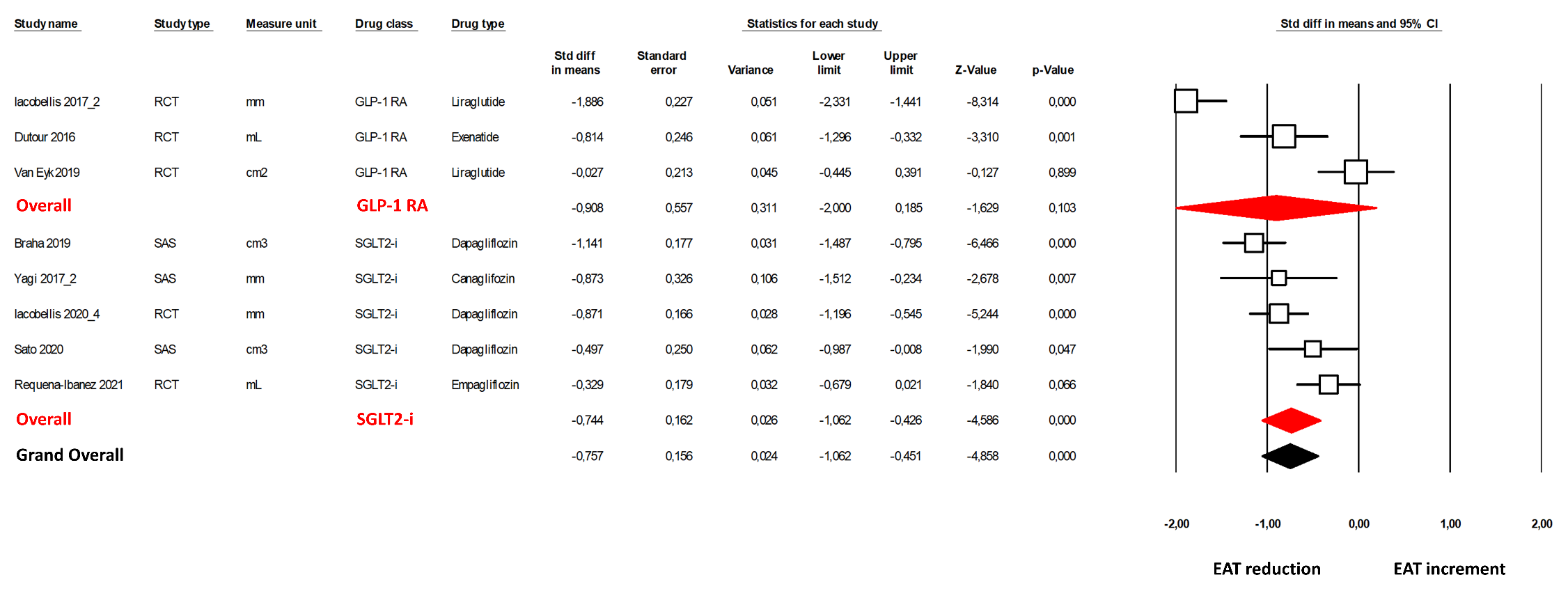


**Figure S2.** Forest plots of the GLP-1 agonists and SGLT-2 inhibitor's effect on EAT reduction within 6 months of follow-up.

**
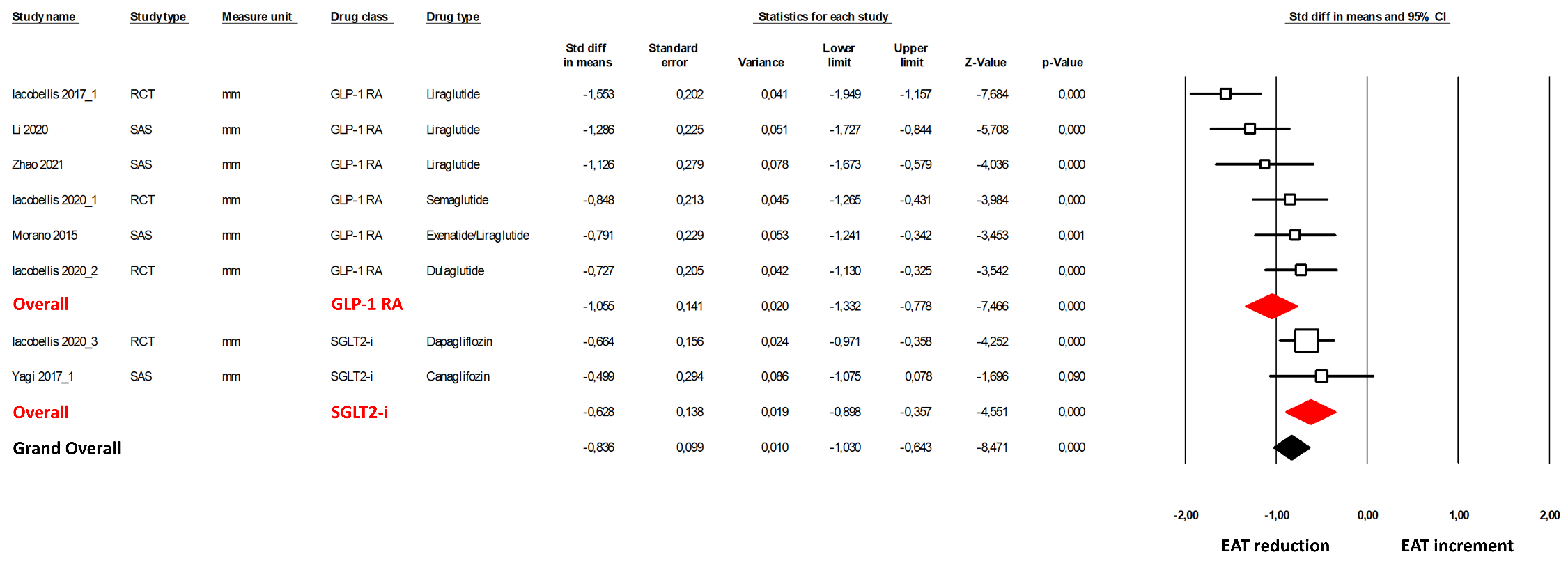
**

**Figure S3.** Forest plots of the GLP-1 agonists and SGLT-2 inhibitor's effect on EAT thickening reduction measured by ultrasound within 3 months of follow-up.


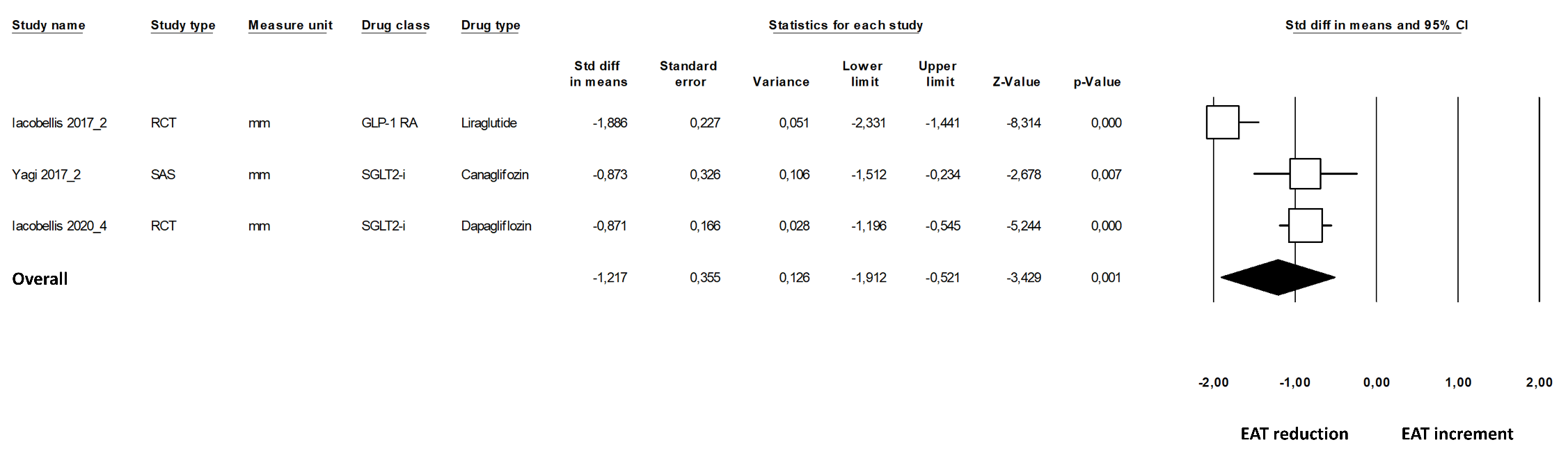


**Figure S4.** Forest plots of the GLP-1 agonists and SGLT-2 inhibitor's effect on epicardia adipose tissue thickening reduction measured by ultrasound within 6 months of follow-up.


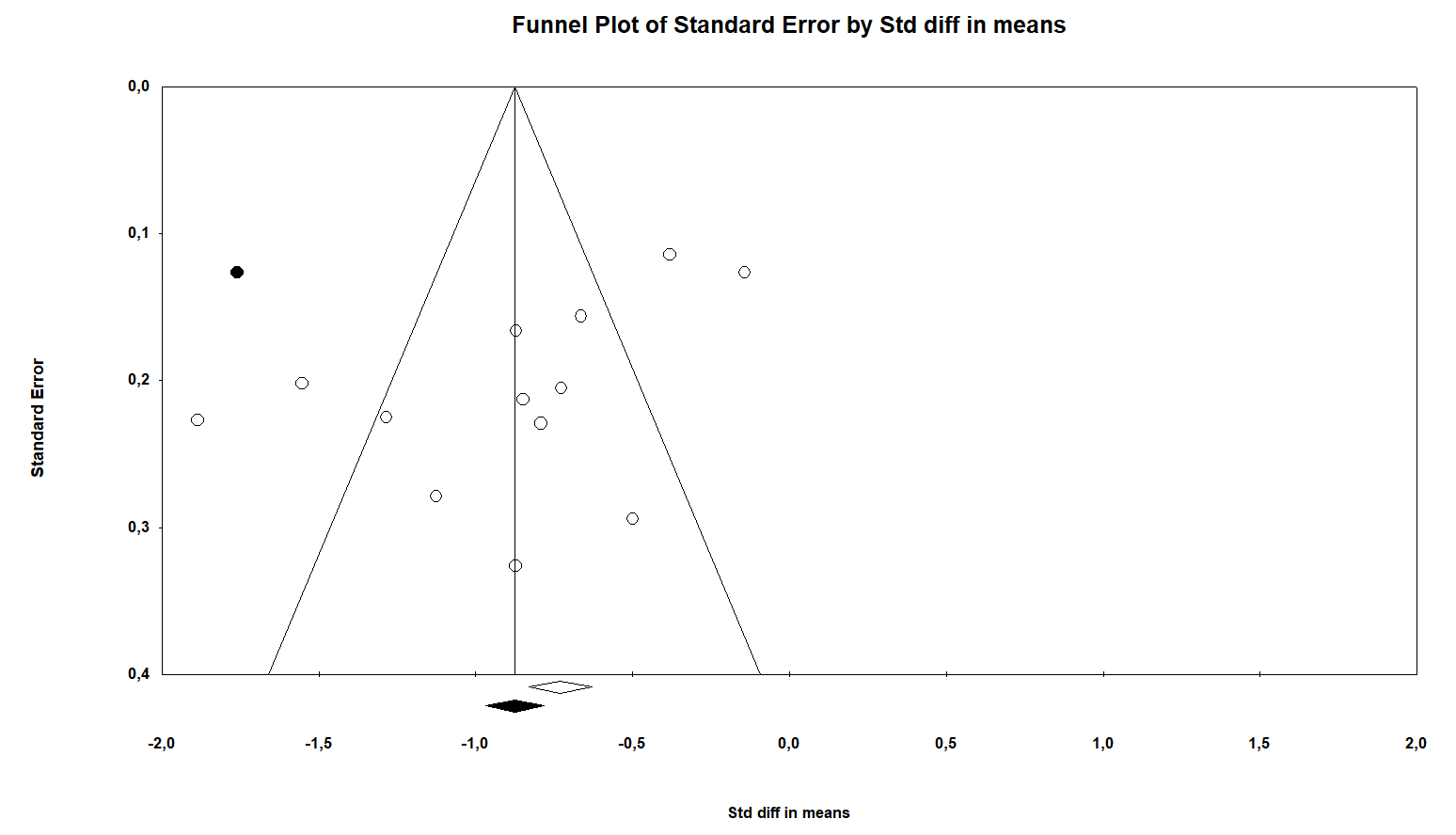


**Figure S5.** Funnel plot of effect size versus standard error for the overall effect of cardiometabolic drugs on EAT thickening. Dots represent the single studies while diamonds are the overall standardized mean difference (Std diff in means). White filling refers to real studies while black fill relates to Duval and Tweedie’s trim and fill method.


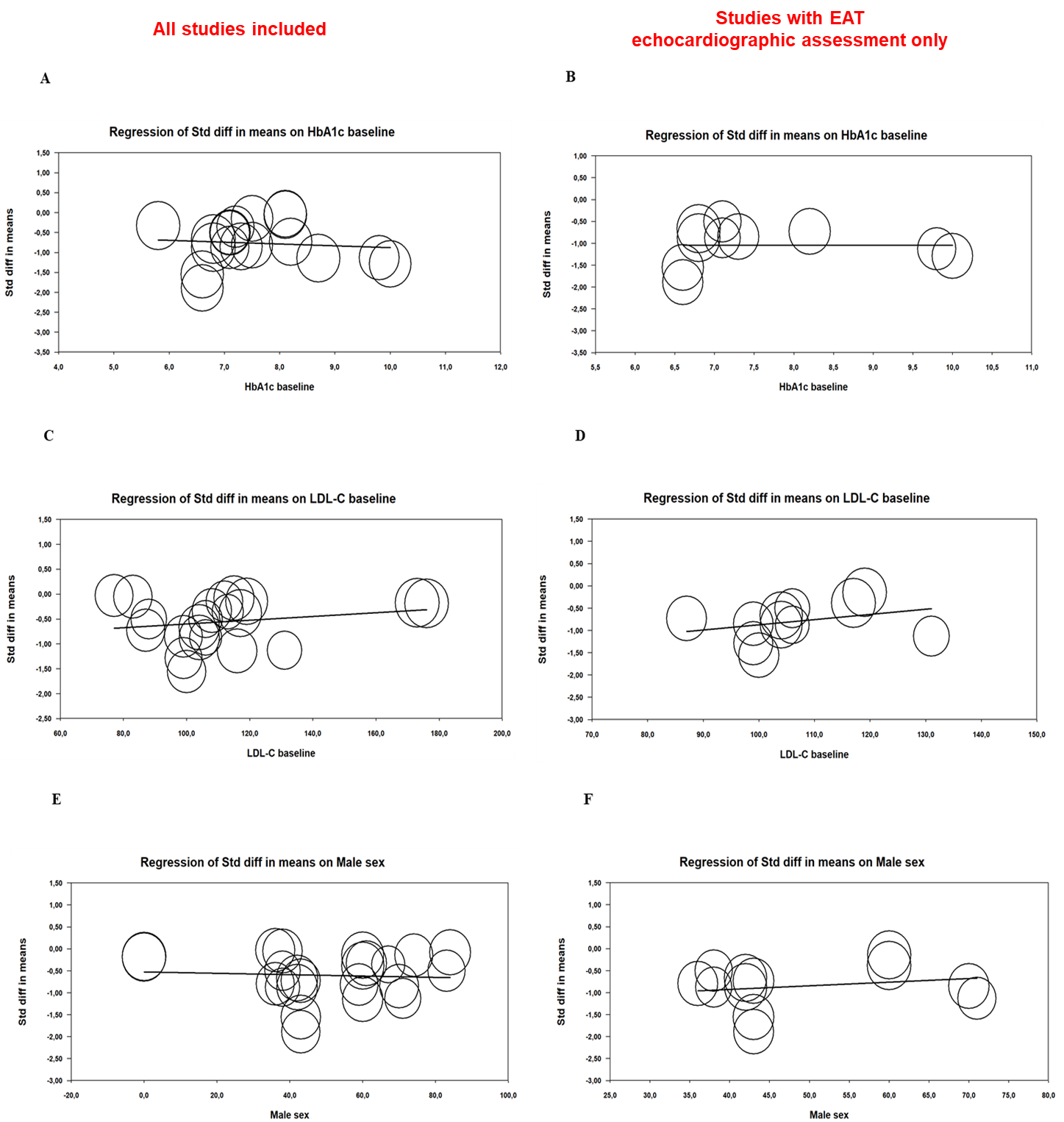


**Figure S6. Meta-regression analysis**. Impact of HbA1c on the difference in composite cardiometabolic drugs effect and EAT reduction (**A**), and cardiometabolic drugs effect and EAT thickness reduction measured ultrasound (**B**). Impact of cholesterol low-density lipoprotein levels (LDL-C) on the difference in composite cardiometabolic drugs effect and EAT reduction (**C**), and cardiometabolic drugs effect and EAT thickness reduction measured ultrasound (**D**). Impact of male sex on the difference in composite cardiometabolic drugs effect and EAT reduction (**E**), and cardiometabolic drugs effect and EAT thickness reduction measured ultrasound (**F**).
